# Developing health systems research capacities through north-south partnership: An evaluation of collaboration with South Africa and Thailand

**DOI:** 10.1186/1478-4505-6-8

**Published:** 2008-08-01

**Authors:** Susannah H Mayhew, Jane Doherty, Siriwan Pitayarangsarit

**Affiliations:** 1 Centre for Population Studies, London School of Hygiene and Tropical Medicine, London, UK; 2 Independent consultant and part-time lecturer, School of Public Health, University of the Witwatersrand, Johannesburg, South Africa; 3 International Health Policy Program, Bureau of Health Policy and Strategy, Ministry of Public Health, Nonthaburi, Thailand

## Abstract

**Background:**

Over the past ten years, calls to strengthen health systems research capacities in low and middle income countries have increased. One mechanism for capacity development is the partnering of northern and southern institutions. However, detailed case-studies of north-south partnerships, at least in the domain of health systems research, remain limited.

This study aims to evaluate the partnerships developed between the Health Economics and Financing Programme of the London School of Hygiene and Tropical Medicine and three research partners in South Africa and Thailand to strengthen health economics-related research capacity.

**Methods:**

Data from programme documents were collected over five years to measure quantitative indicators of capacity development. Qualitative data were obtained from 25 in-depth interviews with programme staff from South Africa, Thailand and London.

**Results and Discussion:**

Five years of formal partnership resulted in substantial strengthening of individual research skills and moderate instituonalised strengthening in southern partner institutions. Activities included joint proposals, research and articles, staff exchange and post-graduate training. In Thailand, individual capacities were built through post-graduate training and the partner institution developed this as part of a package aimed at retaining young researchers at the institution. In South Africa, local post-graduate teaching programs were strengthened, regular staff visits/exchanges initiated and maintained and funding secured for several large-scale, multi-partner projects. These activities could not have been achieved without good personal relationships between members of the partner institutions, built on trust developed over twenty years. In South Africa, a critical factor was the joint appointment of a London staff member on long-term secondment to one of the partner institutions.

**Conclusion:**

As partnerships mature the needs of partners change and new challenges emerge. Partners' differing research priorities (national v international; policy-led v academic-led) need to be balanced and equitable funding mechanisms developed recognising the needs and constraints faced by both southern and northern partners. Institutionalising partnerships (through long-term development of trust, engagement of a broad range of staff in joint activities and joint appointment of staff), and developing responsive mechanisms for governing these partnerships (through regular joint negotiation of research priorities and funding issues), can address these challenges in mutually acceptable ways. Indeed, by late 2005 the partnership under scrutiny in this paper had evolved into a wider consortium involving additional partners, more explicit mechanisms for managing institutional relationships and some core funding for partners. Most importantly, this study has shown that it is possible for long-term north-south partnership commitments to yield fruit and to strengthen the capacities of public health research and training institutions in less developed countries.

## Background

Over the past ten years calls to strengthen and invest in health systems research capacities in low and middle income countries have increased [1-4]. For example, the 2004 Ministerial Summit on Health Research in Mexico called for national governments to increase their expenditure on health research citing the Commission on Health Research for Development (COHRED) recommendation that governments spend 2% of national health budgets and 5% of international development aid for health on research [5]. One mechanism for capacity development is the partnering of northern and southern institutions, and a number of articles and donor documents have suggested strategies for strengthening and evaluating such partnerships [6-10]. However, detailed case-studies of north-south partnerships, at least in the domain of health systems research, remain limited [11-13].

This paper summarises an evaluation of efforts made by the Health Economics and Financing Programme (HEFP) of the London School of Hygiene and Tropical Medicine to strengthen health economics-related research capacities in South Africa and Thailand between 1999 and 2005 [14-16]. HEFP is a research programme funded for the past 15 years by the UK Department for International Development (DFID). HEFP was one of the first wave of research 'Knowledge Programme' grants created by DFID. Initially HEFP was the sole contractor and the programme helped to build up a body of specialist researchers at LSHTM. In 1999 more emphasis was placed on research collaboration and over the years HEFP developed strong partnerships with institutions in South Africa and Thailand with the aim of strengthening their research capacities and producing nationally and internationally relevant knowledge. This paper provides a case-study of these research relationships over time.

We first present findings from the quantitative assessment of indicators and then discuss the qualitative perceptions of the partnership highlighting the successes and challenges of partnership in each country and drawing out lessons for the development of north-south research partnerships for capacity building elsewhere.

### The partners

The specific goal of HEFP is to improve the equity, efficiency and quality of health services in developing countries through the application of health economics. One of the programme's aims is to develop and consolidate strong partnerships with southern institutions in research, teaching and communication of new knowledge. Initially HEFP concentrated on building up a body of specialist researchers in London while developing strong but informal relationships with research institutions in South Africa and Thailand, both middle-income countries. In 1999 research collaboration was formalised and Memoranda of Understanding were signed between HEFP and three southern research partners: in South Africa there was a 3-way MOU between Partner 1, Partner 2 and HEFP; in Thailand the MOU was between Partner 3 and HEFP. These MOUs were almost identical and described explicit commitments based on two joint collaboration goals: 1) to support development of capacity in research, teaching and communicating research and 2) to jointly carry out research that would increase knowledge in five specified areas. Specified activities included: joint research, joint publications, staff exchanges and training, HEFP contributions to teaching, HEFP assistance in accessing sources of funds; putting in joint funding applications; and funding some low-costs activities from Programme funds.

While all the partner organisations were involved in research, in South Africa both partners were based at universities while in Thailand the partner was a government research institution. In both countries the partners were well-established institutions with existing health research capacities and independent sources of funding, though there were significant contextual differences between the partners which shaped the nature of their relationship with London over time.

**Figure 1:**
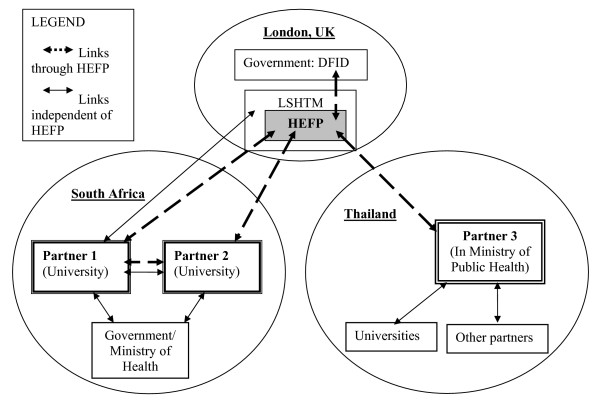
Relationship between HEFP and its core partner institutions.

Figure 1 indicates the relationships between the partners. While the focus of this paper is the links forged through HEFP, the partners also had links with one another, and with the London School, beyond the HEFP partnership.

## Methods

In 2004/05, the authors were requested by the participating institutions to evaluate the partnership during the final year for which they had funding. The evaluation combined quantitative and qualitative data collection and analysis techniques. An evaluation framework was developed that drew on measures and methods of analysis for three strands of capacity strengthening: organisational capacity strengthening [17,18]; research capacity strengthening [4,19,21,22]; and strengthening partnerships [23,24]. Our core indicators are shown in Table 1.

**Table 1:** Indicators for Evaluation

**Areas for Capacity Building**	**Staff Training**	**Learning on-the-job**	**Institutional Partnership**
			
**Level of Indicator**			
**Individual**	# staff trained through: - MSc course - PhD course - workshops/meetings	# 1st authored papers by southern partner # projects led by southern partner *Southern staff initiate project/paper ideas*	*Staff feel there is trust between partners* *Staff feel there is shared decision making*
**Institutional**	Proportion of staff with MSc Proportion of staff with PhD # staff participating in project visits, exchanges & mentoring	Proportion of institutional papers resulting from partnership Proportion of institutional projects resulting from partnership	Northern staff give technical support to strengthen teaching at Southern institution: - teaching - supervision of PhDs - examiner duties - technical input on establishment of MSc/PhD programs *Staff feel they are in partnership*

All indicators are quantitative except those in italics which are qualitative

Quantitative data were obtained from an extensive document analysis of programme memoranda of understanding, annual reports and other programme documentation from each partner for the whole project period. These sources revealed the extent to which the partnership contributed to staff training, projects funded and papers published and identified the tangible, 'measurable' products of the partnerships within the time-frame of the study (1999–2004). Attributing products to the partnership was not without problems, given the shifting of staff between projects, the time-lag between the completion of projects and publication of articles, and the diffuse, long-term influence of the partnership in both its informal and formal stages on the developing research agendas of all partner institutions. Therefore qualitative data were considered equally important.

The qualitative data give us information on the less tangible, but arguably more influential, aspects of partnership such as trust and perceived value. This was obtained through 25 semi-structured stakeholder interviews. Thirteen respondents were from the South African partner institutions, nine were from the Thai partner institution and three were from HEFP. All stakeholders were purposively selected: twenty of them were stakeholders who had been part of the partnership activities for several years; five were policy-makers who were able to comment on the policy-relevance of the partnership. Data were analysed manually using a framework analysis approach to identify the views and attitudes of key stakeholders around the core research themes.

## Results and Discussion

### Strengthening research capacities

The South African and Thai institutions had different needs and capacity-strengthening priorities. The institutional priorities resulted in slightly different activities being undertaken in each country; these are summarised in Table 2.

**Table 2:** Key activities undertaken to build capacity of research partners in South Africa and Thailand

**Key capacity building activities**	**South Africa**		**Thailand**
		
	**Partner 1**	**Partner 2**	**Partner 3**
**Individual Level**			

Joint research projects	X	X	X
Joint publications and dissemination of results	X	X	X
MSc/PhD training in London*	X		X
Support to MSc/PhD training at partner institution	X	X	

**Institutional Level**			

Development/strengthening of teaching programmes	X	X	
Staff secondment	X		
Staff mentor-ship	X	X	X
Staff exchanges	X	X	
Staff project visits	X	X	X
Participation in professional networks strengthened	X	X	X

* One staff member from Partner 2 did pursue a PhD at the London School but this was a once-off occurrence. Partner 2 offered its own MSc in Health Economics and therefore did not send MSc students to London, but Partner 1 sent staff more frequently.

#### Post-graduate training, strengthening of teaching programmes and staff exchanges

The Thais identified the need to train up young staff to ensure the next generation of public health researchers. Being a government institution they could not do this themselves and therefore looked to London's PhD programme, which became the main focus of capacity building (9 PhD students were enrolled during the study period). This was not directly funded though the partnership which meant that a number of Thai staff did not consider the PhD training to have much to do with HEFP. Nevertheless, HEFP staff did contribute to securing PhD funding (through scholarship and project applications) and Thai staff did recognise the wider benefits of training a cadre of staff at the London School since it provided both '*a platform to share experiences and ... a training program for [our] staff' *(Interview, Thai Director). Thus, whereas in 1999 in Thailand only the Director held a PhD, by the end of 2004 three staff held PhDs from the London School and two more staff were about to achieve theirs, representing a strengthening of about 20% of the institution's staff.

In South Africa, both institutions were university-based and, to minimise brain-drain, wanted to strengthen their own capacities for teaching at MSc and PhD levels. Accordingly, London provided technical assistance to strengthen and develop higher-education programmes in South Africa, offering teaching, mentorship of young lecturers, examination of MScs and PhDs and advice on curricula development and teaching procedures. Some young researchers also attended courses in London. Table 3 indicates the number of staff trained in each institution both in London and in South Africa, and the teaching undertaken by London staff to support the South African teaching programs. Table 4 shows numbers of workshops, exchanges and mentorship visits undertaken by staff.

**Table 3:** Staff involved in post-graduate training and teaching activities

**Activity**	**Partner**	**Year 1 (1999–2000)**	**Year 2 (2000–2001)**	**Year 3 (2001–2002)**	**Year 4 (2002–2003)**	**Year 5 (2003–2004)**	**TOTALS**
**Staff trained in research through MSc/PhD programmes (# new staff each year)**	Partner 1	1 MSc student in London	1 MSc student in London	-	-	1 MSc student in London; 1 PhD supervised by HEFP in SA	**3 MSs in London**
							**1 PhD in SA**
	
	Partner 2	-	1 PhD student in London		1 PhD student visits London to refine thesis	-	**4 PhDs (1 in London 3 in SA)**
			2 PhD students supported by HEFP in SA				
	
	Partner 3	4 PhD students in London	1 PhD student in London		2 PhD student in London	2 PhD students in London	**9 PhDs in London (5 complete)**

**London staff involved in MSc teaching/support to South Africa**	Partner 1	3 staff teach on short-course	3 staff teach on short-course	Staff teach on health economics modules	Staff teach on health economics modules	Staff teach on health economics modules	**Multiple staff**
		Staff teach on health economics modules	Staff teach on health economics modules	Staff teach on new policy analysis module	Staff teach on policy analysis module	Staff teach on policy analysis module	
			Staff advise on development of private sector module				
	
	Partner 2	Staff teach on two modules	Staff teach on one module	Staff teach on one module	Staff teach on one module	Staff teach on one module	**Multiple staff**
		Staff acts as external examiner	Staff acts as external examiner	Staff acts as external examiner	Staff acts as external examiner	Staff acts as external examiner	
					Staff examine dissertations	Staff examine dissertations	

**London staff involved in PhD teaching/support to South Africa**	Partner 1				Support to coordinate new PhD program	Support to coordinate PhD program	**Multiple staff**
					PhD supervision	PhD supervision	
						Workshop on PhD supervision	
	
	Partner 2		PhD supervision	PhD supervision	PhD supervision	PhD supervision	**Multiple staff**
						Workshop on PhD supervision	

**Table 4:** Staff involved in key workshop training, mentoring and exchange activities

**Activity**	**Partner**	**Year 1 (1999–2000)**	**Year 2 (2000–2001)**	**Year 3 (2001–2002)**	**Year 4 (2002–2003)**	**Year 5 (2003–2004)**	**TOTALS**
**Staff training through workshops/meetings**	Partner 1	1 staff to London	c.2 staff attend London workshop	c.4 staff attend workshop by HEFP in SA	c.2 staff to London	-	**9 SA staff**
	
	Partner 2	3 staff to London	c.2 staff attend London workshop	c.4 staff attend workshop by HEFP in SA	c.3 staff to London	-	**12 SA staff**
	
	Partner 3	-	5 staff attend London workshop	-	-	3 staff attend London workshop	**8 Thai staff**

**Staff exchanges, 'mentorships' and project visits**	Partner 1	3 staff to London	5 staff to London	2 staff to London	5 staff to London	5 staff to London	**37 staff (20 to London; 17 to SA)**
		5 HEFP staff visit	HEFP Director + 6 other staff visit	1 HEFP staff visits	2 HEFP staff visit	2 HEFP staff visit	
	
	Partner 2	1 staff to London	2 staff to London	1 staff to London	2 staff to London	3 HEFP staff visit	**22 staff (6 to London; 16 to SA)**
		4 HEFP staff visit	HEFP Director + 3 other staff visit	3 HEFP staff visits	2 HEFP staff visit		
	
	Partner 3	5 staff to London	HEFP Director visits	Director to London	2 staff to London	1 staff to London	**15 staff (9 to London; 6 to Thailand)**
		HEFP Director visits			HEFP Director visits Thailand twice; 2 other HEFP staff visit	HEFP Director visits	

All in all, PhD training contributed significantly to enhancing the advanced research capacities of all partners, particularly the Thais, and the proportion of staff trained in post-graduate research skills at each institution increased between the beginning and end of the study period.

Over and above post-graduate training, familiarity with London staff, their research and teaching procedures and interaction with the international research community was facilitated by frequent exchanges, mentorships and PhD-related periods spent in London. The greater exchange and contact between the London and South African staff that flowed from the engagement between the teaching programs at the three universities resulted in a greater feeling of partnership than with the Thais for whom contact was either during PhD training or between institutional directors rather than lower-level staff (Table 4).

#### Joint projects: learning and partnership through doing

All partners were interested in implementing joint projects on topics of mutual interest and in jointly disseminating this new knowledge: this became a core component of collaboration (see Table 5). The partnership arrangement with HEFP did not, in itself, provide research funds, but the involvement of HEFP in joint proposals improved the likelihood of southern institutions receiving funding.

**Table 5:** Number and % joint projects and peer-reviewed publications by country and lead institute and place of publication

**SOUTH AFRICA**	**1999/2000**	**2000/2001**	**2001/2002**	**2002/2003**	**2003/2004**	**Partnership Totals**
***New joint projects funded: #***	**6**	**4**	**3**	**3**	**2**	**18**
P1: Lead	2	2 joint	1	1 joint	0	6
Involved	6	2	2	1	2	13
P2: Lead	1	0	0	1	0	2
Involved	3	3	2	3	1	12
HEFP: Lead	3	2 + 2 joint	2	1 + 1 joint	2	10
Involved	6	4	3	3	2	18
***Published articles relating to link: *#**	**6**	**6**	**16**	**11**	**18**	**57**
Institutional affiliation of first author:* P1**	2	0	11	3	10	22
P2	0	3	3	3	1	10
HEFP**	1	1	1	2	4	9
Jointly appointed HEFP/P1 staff member based at P1	3	2	2	2	2	11
**THAILAND**						
**New joint projects funded: #**	**1**	**3**	**2**	**2**	**2**	**10**
Institutional lead: P3	1	2	1	2	2	8
HEFP	0	1	1	0	0	2
***Published articles relating to link: *#**	**2**	**1**	**1**	**3**	**7**	**14**
Institutional affiliation of first author:* P3	2	0	1	3	6	12
HEFP	0	1	0	0	1	2

* article excluded if first author from an outside institution

** excludes publications 1^st ^authored by staff member jointly appointed to HEFP/P1

P1 = Partner 1; P2 = Partner 2; P3 = Partner 3.

Collaborative development and implementation of projects was achieved through regular email contact and face-to-face meetings, visits and workshops (Table 4) to facilitate joint assessment of progress, discussion of analyses and report writing.

In Thailand, eight of ten funded projects were for PhD research with only two being broader, multi-staff research projects. This lack of large joint projects is reflected in the lower levels of staff exchange (Table 4) and meant that Thai staff reported more mixed benefits of the partnership. Language was also identified as a difficulty, especially for qualitative projects and publications as, unlike in South Africa, English is not the lingua franca in Thailand. Not surprisingly, with most projects relating to PhDs, the income from 'partnership' projects was only a small proportion of Partner 3's total income, though it increased between 1999 and 2004 as the number of funded PhD projects increased.

In South Africa, by contrast, 18 projects (Table 5) were developed and implemented in the six years of formal partnership and this collaboration was seen by respondents there as taking people forward *'by leaps and bounds'*. Some of these joint projects were financially large and contributed significantly to institutional income.

Probably the most influential factor in both individual and institutional capacity building for research in South Africa was the full-time, long-term secondment, on a jointly funded post, of an HEFP staff member to South African Partner One. This person also had responsibilities towards Partner Two. Having a dedicated link person has enabled provision of on-the-ground guidance and support on a wide range of issues relating to research. The individual is recognised by South African and London respondents as '*phenomenally important*', putting a huge *'level of energy into capacity building' *and credited with ensuring that the work produced by the institution '*has been of international standard*.' This has resulted in particularly close relationships developing between HEFP and Partner One which saw the development of more projects, more joint publications and more joint activities than between any of the other partners (Tables 3, 4 and 5).

#### Joint Publications

The proportion of southern-first-authored, peer-reviewed articles was already high at the beginning of the partnership, but the number of papers increased over the partnership and first authorship levels were maintained (Table 5). This reflects the high level of project collaboration, especially in Partner One. In most of the years of the partnership very few peer-reviewed articles were first-authored by a junior researcher from a partner institution, PhD-related papers being an exception (however, the number of reports and conference papers first-authored by junior researchers was higher). Adequate support to junior staff interested in first-authoring papers remained a challenge for all partners, including London, although all partners made explicit efforts to try to address this through writing workshops and mentorship.

### Lessons and Challenges of Partnerships

#### Institutionalising trust

The partnerships with South Africa and with Thailand both had their roots in relationships forged through individuals who undertook post-graduate training at the London School and then went on to play key roles in the partner institutions. These links with individuals go back to the mid-1980s and formed the basis for the evolving trust between the different institutions and for joint activities in these particular countries:

'*Personal relationships were critical at the beginning ... that's what makes the collaborations work – the long relationships*.' (Interview, London staff)

Important factors that connected and built on the personal links were the shared interests and ideology on research-related issues that were held by a wider group of people in the two countries and allowed the expansion of a partnership beyond the individuals at its heart. Thus, when the partnership was formally consolidated in 1999, it was seen by the respondents as the culmination of a naturally evolving relationship, rather than as something new and imposed.

Nonetheless, the institutionalisation of the partnership was more successful in South Africa (especially with Partner One) than in Thailand. First, the shared language between South Africa and the UK made it easier for staff to engage with one another. Second, the creation of a joint post for an HEFP staff member in Partner One played a critical role in the development of a broader institutional partnership that also drew in Partner Two. The individual – who has spent more than ten years in South Africa – proactively engaged a broad range of people in partnership activities and helped to secure British Council funding to facilitate further staff exchanges (including of administrative staff). The extensive interaction of junior staff through mentorships and exchanges helped a young cadre of staff to feel engaged in the partnership and built trustful relationships with London staff through joint working and support. Furthermore, other senior members of Partner One developed independent relationships – and associated research projects – with other components of the London School over time. Despite these successes, respondents emphasised the need to continue searching for mechanisms to enhance the institutional, rather than personal, aspects of the partnership.

In Thailand, the staff regard the partnership as still primarily a personal link between the Directors of the Thai partner and HEFP; a perception exacerbated by the culture of hierarchy in Thailand (only gradually changing) which is manifest in the particular management style of the institution's director:

*'Few [institution] members know the details about the network and most information is held by [the Director] ... he is quite busy and may feel it unnecessary to provide the information regarding the collaboration.' *(Interview, Thai staff)

In recognition of this, the Thai institution set up a management committee in May 2004 to involve more staff in assisting the Director in management decisions including the HEFP link. To some extent the lack of joint projects (other than PhD work) has limited the opportunities for joint working. This, together with differing research priorities between Partner 3 and HEFP (further discussed under 'Equity in North-South Relationship' below), made it more difficult to build relationships of 'trust' with the younger Thai staff members, some of whom who felt that London was more interested in its own research priorities than in theirs. Moreover the primary partnership activity focus on PhD training perpetuated a feeling of unequal relationships since London staff were 'teachers' and Thai staff were 'students', though in Thailand they had a much higher professional profile. This helped to contribute to a feeling of disengagement or unequal partnership below the level of the Director at the Thai institution. The development of post-doc collaborative research projects is therefore highlighted by the Thai partners as a priority.

#### Sustaining capacity

Southern partners typically face a number of challenges in sustaining strengthened research capacity. These include factors related to the context of the wider system in which the institution operates, in particular the competition that the institution faces [25]. In this study, the biggest institutional challenge identified by both partner countries was the lack of incentives and career structures for staff, particularly junior staff, who may find more attractive offers in clinical practice, government, the private sector, or abroad – this is particularly so for skilled black professionals in South Africa for whom there is an increasingly competitive market.

Partners in both countries developed strategies to help retain their staff, with some degree of success. Training, facilitated by the HEFP link, was one strategy: investing in PhD training was considered important not only for building internal capacity but also as a career progression incentive that helped retain staff, at least in the medium term.

In South Africa, although both universities lost some trained staff, these all remained working in South Africa: this can be considered positive for building wider national research capacity:

*'... there is always a disappointment in [the South African institutions] that they churned out people with research skills who then went into government or the private sector ... my view is, that's fine – isn't that what you want? People capable of doing research?*' (Interview, government official, South Africa)

In Thailand, where the partner institution is a quasi-government institution, research staff are technically seconded from the Ministry of Public Health which does not recognise 'research' as a 'career'. One interim solution that the Thais implemented was to 'top-up' salaries equivalent to promotion from project grants. Moreover, the Director of the institution had a strong reputation which attracted staff. Beyond that, he was committed to the long-term training of staff specialised in health systems and economics research and developed a prestigious training package to attract recruits, offering '*fantastic support to their staff*" (Interview, London staff). This includes several years of training – both in the form of PhD training in London and on-the-job training within the institution – as well as support to attend international conferences and workshops, with staff often shadowing official Thai delegations.

#### Equity in North-South relationships: negotiating policy-relevant research priorities and finances

##### Negotiating policy-relevant research priorities

To some extent the partners have different research priorities. HEFP is concerned with research of international rather than national relevance, with a strong emphasis on cross-country comparison driven by donor pressure for 'international knowledge' (Interview, London staff). Thai research priorities were driven largely by their national and policy commitments, often requiring work to be done far more quickly than allowed for by the slow process of research proposal development and fund-raising:

*'Most work we are interested in is ... not a research question, but more how to answer the questions quickly to the Minister – there is no academic challenge in this.' *(Interivew, Thai staff)

In many ways the Thais' policy-led research has had more direct impact on policy e.g. health care costing and budget estimation exercises for Universal Coverage demonstrated the feasibility of the policy to policy-makers so that the policy could be implemented rapidly. The more academic HEFP projects, on the other hand, were seen as '*part of a jigsaw that helps to build up a bigger picture' *(Interview, Thai staff).

In South Africa, the early years of partnership with HEFP through the sharing of common academic interests resulted in a number of very strong, long-term projects with HEFP that involved joint proposal development, data collection and analysis – these benefited both HEFP and the South African partners in terms of expanding their research portfolios and bringing in funding to further the partnerships through joint work. Although both university partners in South Africa were committed to policy-relevant research and had good contacts within government, respondents had mixed views on the direct local policy impact of the partnership's research. One of the government officials interviewed felt that the sort of research work that government policy makers required was *'a lot of menial stuff that it is difficult to contract out to universities'*, for example detailed costing of hospitals. The regional and international policy impact of the partnership's research in South Africa was more obvious, however, through its contributions to regional and international networks.

The challenge for the partnership has been how to marry a spectrum of interests that range from international research (London), national and regional research (South Africa) and work for national policy (Thailand). Currently, the London School provides a 'brokering role' by '*facilitating the exchange [between countries] and feeding back country level information to the international community and donors*' (Interview, London staff). Nevertheless, these different priorities lead to different types and levels of academic input that need to be worked into future partnership structures so that they become mutually enhancing.

##### Financing

Financially, all partners receive substantial funds from elsewhere, including international and national bodies. The South African partners generally considered HEFP to have been very important in improving access to funding. In Thailand, where large joint projects were not funded, HEFP's financial contribution was considered relatively insignificant, though the partnership certainly contributed to securing some of the PhD sponsorship. There was a feeling that partnerships would be more equal '*... if we were on joint financial proposals to tap funding rather than just being part of the study and requested to do the research that London wants*...' (Interview, former Thai Director).

For HEFP having consolidated partnerships with southern partners was a major benefit for attracting funding for joint projects, but the increasing financial needs of southern partners are likely to place additional pressure on HEFP who are also facing a less favourable funding climate for European public health research institutions:

'*Both the partners are quite strong ... we struggle to get funding more than they do now. I'd like to see them put us on a proposal that they write, because southern institutions are more likely to get funding*.' (Interview, HEFP staff)

##### South-South relationships to build 'partnership'

This emerged as something each of the southern partners would have liked to have seen but which the HEFP partnership did not explicitly address (as Figure 1 shows). While there was exchange between Partners 1 and 2 because of independent linkages as well as the joint appointee (who, although based at Partner 1, also had official responsibilities towards Partner 2), there was no sustained engagement between Partner 3 and either South African partner. This was largely because of a lack of finances and structures to support such a linkage:

'*If we are to see greater links with others under HEFP, they need to fund south-south collaboration. HEFP never provided support to build a network with other countries directly*...' (Director, Partner 3)

HEFP could probably have done more to foster and encourage south-south working between its own partners but, to be fair, southern partners could also have been more proactive in seeking funding for more engagement with one another. At the time, however, a greater priority for the southern partners tended to be establishing stronger networks within their own regions. Moreover, HEFP, in its role as the broker of the partnership, was caught up in efforts to make the partnership work collectively:

'*... there's not enough time or space to develop partnerships ... we need to factor in time for the consortium which in the past has been managed through personal links. ...we need a structure that is mutually engaging*...' (Director HEFP)

It can be argued that the south-south partnerships that existed *within *South Africa did support a more collegial feeling between the partners and perhaps increased the strength of their voices within the partnership. It is possible therefore that more direct contact between the South Africans and the Thais would have been productive for all southern partners as well as the wider partnership, albeit that the challenges associated with the different research versus government-led priorities would have continued. Greater south-south linkage could have facilitated the move beyond 'collaboration' to a real 'partnership':

*'If it's a collaborative network, then you collaborate on specific activities that have been planned. If it's a partnership, you're not only thinking about the people within the one institution, you're thinking about the entire partnership.' *(South African respondent)

This type of partnership had been achieved by the South African partners with London directly, as another South African respondent notes: '*It's always been, "What are your views? What are your efforts? What do you think of this idea? That has been absolutely impeccable*.' But this type of partnership between the southern partners was not developed because '*everything was managed through London*' (Partner 3 staff).

## Conclusion

This study has shown that five years of formal partnership have resulted in significant strengthening of individual research skills and moderate institutionalised strengthening in southern partner institutions. For HEFP the benefits of partnership were multiple – from gaining access to interesting research topics and case-studies in southern countries, helping to attract funding for joint work to gaining an entry-point for influence in national as well as international policy. Joint project work in South Africa was extremely successful with many joint projects funded. These provided opportunities for a broad range of staff involvement and considerable management and capacity support as well as research guidance from HEFP, particularly through the staff secondment. An impressive number of publications followed together with international recognition of the value of the research. In Thailand the focus on PhD work, the lack of funding attracted for other joint work, and language difficulties, meant that the broad-based involvement of staff and production of papers was considerably less.

The extensive PhD training programme engaged in by Thailand was considered very valuable in developing a cadre of competent health economics and policy research staff and giving young researchers international exposure through studying abroad. In South Africa strong support was given for the university units to develop and expand their own PhD and masters programmes, though the opportunities this offers for two-way teaching staff exchange with London have not yet been exploited.

For all southern partners, pre-existing research capacity increased the likelihood of successful partnership. Over and above this, however, the activities described above could not have been achieved without good personal relationships between members of the partner institutions, built on trust developed over twenty years. In South Africa, a critical factor was the joint appointment of a London staff member on long-term secondment to one of the partner institutions – something donors have, for decades, been reluctant to fund. Trust is built slowly through long-term commitment to funding and joint working through staff exchanges, funding for joint meetings to develop research proposals around common interests, analyse data, write up and disseminate results together. These things need to be undertaken in a way that involves southern partners as equal members of the partnership, adapting goals and priorities according to collective, not northern-led, interests. As discussed, this proved more challenging in Thailand which had direct, short-term policy-relevant priorities, than with the South African academic institutions which shared a more similar long-term, exploratory approach to research.

Marrying the different research interests and priorities of the different partners was one of the biggest challenges to the partnership. In South Africa, the academic institutions shared similar goals, priorities, organisational structures and constraints to those faced by the Northern partner. The extensive programme of staff exchange through teaching at different staff-levels as well as the joint appointment gave staff in partners 1 and 2 a greater feeling of partnership than was possible in Thailand where the partnership was not institutionalised in the same way. The immediate policy-needs of the Thai partner meant staff often could not invest in longer-term research projects; its staff were government employees not academic staff so staff exchanges were not as 'equals' in the sense they were in South Africa. Moreover, the personal contacts and 'partnership' was largely concentrated with the Director, giving less feeling of engagement in the organisation as a whole.

It is hard to say whether one type of partner was more 'productive' than the other because each was productive in a different way. The South African academic institutions brought in more research money and published more academic papers, but the Thai partner had more direct impact on national policy. In terms of whether the partnerships achieved HEFP's objectives, it could be said that the overarching goal of HEFP, to improve services through the application of health economics, may have been better met with more immediacy in Thailand where direct policy influence was achieved. The training of researchers in South Africa who then went to government institutions was also an important contribution to national capacity-building in economics research but it was not possible to quantify their direct impact on research. On the other hand, the aim of HEFP, to strengthen partnerships in research, teaching and communication, was probably better achieved in South Africa where the partners' own goals, priorities and structures were more similar.

Both types of partnership, and achievement, are valuable and the northern partner's biggest lesson was probably in how to manage these very different types of partners within the same partnership. This entailed giving the space and flexibility to each of the different partners to define their own national priorities while seeking to engage each partner in a wider understanding of, and contribution to, international research interests. The major lessons learned were perhaps the need to build in time for the 'consortium' as a whole in order to enhance partnership structures, participation and discussion on research and policy priorities and encourage south-south linkage. The partnership now seeks to scale-up currently successful activities while also acting on these lessons.

Our study has also illustrated how the needs of partners change as partnerships mature and new challenges emerge. Partners' differing research priorities need to be balanced between international and national relevance and research-driven and policy-driven concerns. As the funding climate becomes more difficult for northern public health institutions, and southern institutions expand their research portfolios, southern partners come under pressure to broaden their own funding base rather than rely on northern partners. The development of mutually engaging partnership structures will ensure that these changing needs enhance, rather than weaken, the partnership.

The type of capacity building discussed in this paper cannot be achieved without substantial financial inputs. Much of the specific capacity strengthening in these partnerships was done on the back of research funding and it is therefore hard to put a precise cost-estimate to it. Much of what was achieved was possible because the partners were in middle-income settings. In low-income countries building capacity through this type of partnership and joint research is extremely difficult, yet donors, while calling for broader research partnerships as in DFID's new Research Programme Consortia, do not commit adequate funds. If donors genuinely wish to support successful research capacity building and partnerships then they must commit to long-term and in-country support of the nature described here.

Institutionalising partnerships (through long-term development of trust, engagement of a broad range of staff in joint activities and joint-appointment of staff), and developing responsive mechanisms for governing these partnerships (through regular joint negotiation of research priorities and funding issues), can address these challenges in mutually acceptable ways. Indeed, by late 2005 the partnership under scrutiny in this paper had evolved into a wider consortium involving additional partners, more explicit mechanisms for managing institutional relationships and some core funding for partners. Most importantly, this study has shown that it is possible for long-term north-south partnership commitments to yield fruit and to strengthen the capacities of public health research and training institutions in less developed countries.

## Competing interests

Non-financial

Mayhew works at the London School, but not at the unit running the programme under evaluation. Doherty works at Wits University and formerly was a staff member at Partner 1, while Pitayarangsarit still works at Partner 3, giving them an 'insider' status: this insider status was seen as contributing to the validity of the evaluation as they had in-depth knowledge of the partners and were trusted by them. None of the authors was involved in the programme while it was being evaluated. Partners were afforded the opportunity of commenting on the country reports, and an earlier version of this paper, to check that none of their views had been misrepresented.

Financial

DFID funds HEFP and also funded the evaluation of it, however they had no influence on the design, implementation or analysis of the evaluation. All views in this paper are exclusively those of the authors and do not represent DFID views in any way.

## Authors' contributions

SHM designed and coordinated the evaluation, managed two country-consultants who undertook country studies, undertook primary interviews in Thailand and London, reviewed London programme documents, wrote the Evaluation Comparative Report and prepared the journal manuscript. JD contributed to the evaluation design, undertook the South Africa country study, produced the South Africa Country Report, reviewed South Africa programme documents, undertook qualitative interviews with South African staff, and reviewed and edited the journal manuscript. SP contributed to the evaluation design, undertook the Thailand country study, produced the Thai Country Report, reviewed Thai programme documents, undertook qualitative interviews with Thai staff, and reviewed and commented on the journal manuscript. All authors read and approved the final manuscript.

## Consent

Not applicable: interviewees were not patients; they had been part of the Programme under review and were interviewed in their professional capacities.
